# Construct Validity and Reliability of the Children Participation Assessment Scale-Child version in Children with Physical Disabilities

**DOI:** 10.22037/ijcn.v15i3.26089

**Published:** 2021

**Authors:** Omid ROSTAMZADEH, Malek AMINI, Afsoon HASSANI MEHRBAN

**Affiliations:** 1 Occupational Therapy Department, School of Rehabilitation Sciences, Iran University of Medical Sciences, Tehran, Iran.

**Keywords:** Participation, Occupation, Children, Cerebral palsy, Reliability

## Abstract

**Objective:**

This study was conducted to determine the construct validity and reliability of the Children Participation Assessment Scale in activities outside of School–Child version (CPAS-C) in 6-12-year-old children with physical disabilities (PDs).

**Materials & Methods:**

In this methodological study, participants were 100 children with PDs, recruited from a school for exceptional children with physical-motor disabilities and 100 normally developing children. For assessing the test-retest reliability (ICC), 40 children with PDs completed CPAS-C within a two-week interval, and for assessing the internal consistency (Cronbach's alpha) and construct validity, 100 children with PDs separately completed the Vinland Adaptive Behavioral Scale (VABS) and CPAS-C.

**Result:**

The majority of participants were children with CP, among whom the highest and lowest ratios were related to diplegia (32%) and dystonia (1%), respectively. The results showed that CPAS-C had acceptable reliability (ICC: 0.6-0.99). Cronbach’s α score was between weak to moderate (α = 0.25-0.75). The difference in the score of participation between the two groups (normally developing children and children with physical disabilities) was significant in all areas (P<0.001).

**Conclusion:**

The CPAS-C had acceptable psychometric properties; it can be used as a valid and reliable tool for assessing the participation of 6-12-year-old children with PDs in school activities.

## Introduction

Physical disability is a limitation in physical functioning, mobility, dexterity, or endurance, which results in impaired body functions and structures, as well as reduced activities and participation in tasks (1). Children with physical disabilities, including cerebral palsy (CP), muscular dystrophy, etc., due to the motor and other associated disorders, have limitations in participation in physical activities, play, leisure, and educational school activities (2). Studies show that children with physical disabilities participate less in various activities than their normal peers (3-5). Also, their activities have limited variability, and they are more likely to participate in home activities and the tasks that are less physically and socially engaging (6-8). Children with physical disabilities are at risk of occupational deprivation (9, 10). According to studies, personal (i.e., age, gender, and executive skills) and environmental factors affect the pattern of children’s participation in activities (11). Therefore, the pattern of participation can vary from country to country (5). Since studies have proven that culture has an impact on the patterns of children's participation, it is necessary to assess the participation of children with physical disabilities in activities in various cultures (12). The Occupational Therapy Practice Framework-Third Edition (OTPF-3), published by the American Occupational Therapy Association (AOTA), presents occupations in eight areas of Activities of Daily Living (ADL), Instrumental Activities of Daily Living (IADL), play, leisure, social participation, education, work, and sleep/rest. Children with physical disabilities often have less participation in all activities (5, 13) and experience more problems (14) compared to children with no physical disabilities. Although most assessment tools such as the Children Assessment of Participation and Enjoyment (CAPE) and Children Participation Questionnaire (CPQ) have been developed by occupational therapists, they do not cover all occupational areas (12, 15). The Children’s Participation Assessment Scale- Parent/Child versions (CPAS-P/C) were developed by Amini et al. (2016) in Iran, and the psychometric properties of this scale have been confirmed in normally developing children. This scale covers all the areas of occupation (ADL, IADL, play, leisure, social participation, education, work, and sleep/rest) based on OTPF-3 (16, 17). The child’s report version can provide a reliable answer to the subjective dimensions of participation, such as the child’s enjoyment of different activities (18). Therefore, this study aimed to assess the construct validity and reliability of the Children Participation Assessment Scale-Child version (CPAS-C) among the children with physical disabilities aged 6 to 12 years old.

## Materials & Methods

This study was an instrumental and methodological survey supported and approved by Iran University of Medical Sciences (IR.IUMS.REC 1396.9511355006).

The study population included 6-12-year-old children with physical disabilities and their normal peers of the same age, recruited by the convenience sampling method. The sample size for test-retest reliability was 40, and to assess the internal consistency and construct validity, 100 children with physical disabilities and 100 normally developing children, attending special children’s and ordinary schools in four regions of Tehran (i.e., north, south, west, and east) were enrolled (19, 20). Inclusion criteria for children with physical disabilities were fluency in Farsi, age of 6 to 12 years, and the lack of cognitive problems. 

The children who met the inclusion criteria were screened, and informed consent was obtained from their parents. Two instruments were used in this study. The first was an instrument which was completed by interviewing parents and included a demographic questionnaire, the SPARCLE questionnaire, and Vineland Adaptive Behavior Scale (VABS). The second tool was the CPAS-C scale which was completed by children under the supervision of the examiner (21). Before completing the questionnaires, complete explanations were given on how to complete them, and they were filled out under the supervision of the examiner. 

**Outcome Measurement**

**Child Participation Assessment Scale in Activities Outside of School**

This scale was used to collect information about the participation of children with physical disabilities in occupational performance areas. The CPAS was developed by Amini et al. (2016) in two versions, including the child report and parent report, which the former was used in the present study (i.e., CPAS-C). This scale covers eight occupation areas (ADL, IADL, play, leisure, social participation, education, work, and sleep/rest). This scale examines activities in terms of diversity, frequency, participation, and enjoyment dimensions over the past four months. The time required to complete CPAS-C is 45 to 60 minutes. In typically developing children, the internal consistency of these dimensions varies from 0.83 to 0.85, and the test-retest reliability was reported to be 0.91-0.93 (16).

**Vinland Adaptive Behavior Scale**

Vinland Adaptive Behavior Scale (VABS) is a parent-report scale that is completed by semi-structured interviews and measures the adaptive behaviors of children from birth to the age of 18 in the four dimensions of daily life activities, communication, motor skills, and socialization. In this study, VABS was selected for convergent validity since it was the only tool evaluating the dimensions which were similar to those of CPAS-C. In fact, VABS is commonly used in narrative studies and compilation tools for participatory assessments (22, 23). The overall internal consistency of the Persian version of VABS was reported to be 0.98 (24). In this study, the items related to the ADL, communication, and socialization of 6-12-year-old children were investigated.

**SPARCLE Questionnaire**

This questionnaire is completed by parents to estimate the cognitive level of children with CP. In the present study, children with a cognitive level of over 70 were included.

**Statistical Analysis**

SPSS software version 20 was used for data analysis. For the internal consistency and test-retest reliability, the Cronbach’s alpha and Intraclass Correlation Coefficient (ICC) were used. Also, Pearson correlation was used to assess convergent validity, and the Independent t-test was used to compare the participation scores of children with physical disabilities with those of their normal peers. 

## Results

The age of children with PDs and their normal peers was between 6 and 12 years old with a mean age of 8.83 (SD=1.76) years. Of children with PDs, 56% were boys, and 44% were girls, while these ratios were 54% and 46% for healthy children, respectively.

Out of 100 participants with PDs, 74 had CP. On the other hand, 8, 4, 4, 3, 3, and 4 cases were diagnosed with delayed motor functions, muscular dystrophy, head injury, metabolic diseases, spinal cord injury, and other disorders, respectively. Of the 74 participants with CP, the most had diplegia CP (32%), while the least suffered from dystonia CP (1%). 

The results showed that CPAS-C had acceptable test-retest reliability (ICC: 0.6-0.99) (Table 1). The Cronbach’s alpha score of all occupation areas, except for the two areas of work and sleep/rest, ranged from 0.25 to 0.79 (Table 1). Regarding Pearson correlation coefficients between CPAS-C and VABS scales, the ADL, IADL, and social participation domains of the diversity dimension were not significantly correlated with their three respective VABS domains. However, there was a significant correlation between the IADL domain of CPAS-C and the IADL domain of VABS (P< 0.05, Table 2). Participation scores in all occupation areas were significantly different between normally developing children and those with CP (P <0.001, Table 3). 

**Table1:** Cronbach's alpha (α) and Test-retest reliability (ICC) of CPAS-C

Test-retest reliability (ICC) (n=40)	Cronbach's alpha (α) (N=100)	Dimensions	Occupations
0.86	0.56	diversity	ADL
0.85	0.53	frequency	
0.77	0.48	With whom	
0.93	0.79	enjoyment	
0.99	0.67	diversity	IADL
0.98	0.74	frequency	
0.93	0.50	With whom	
0.98	0.71	enjoyment	
0.98	0.54	diversity	Play
0.98	0.76	frequency	
0.95	0.27	With whom	
0.98	0.58	Enjoyment	
0.98	0.61	Diversity	Leisure
0.97	0.71	frequency	
0.91	0.40	With whom	
0.96	0.66	enjoyment	
0.98	0.62	diversity	Social participation
0.94	0.64	frequency	
0.90	0.46	With whom	
0.97	0.65	Enjoyment	
0.94	0.33	Diversity	Education
0.93	0.25	frequency	
0.94	0.34	With whom	
0.96	0.34	enjoyment	
0.87	0.06	diversity	Work
0.81	-0.11	frequency	
0.87	0.26	With whom	
0.92	0.40	Enjoyment	
0.87	-0.12	Diversity	Sleep/rest
0.88	-0.10	frequency	
0.60	0.20	With whom	
0.91	0.00	Enjoyment	

**Table2:** convergent validity of CPAS-C with VABS

Significance level	(r)	Dimension	CPAS-C domains	VABS domains
0.51	-0. 066	Diversity	ADL	communication
0.49	0.069	Diversity	IADL	
0.81	-0.024	Diversity	Social participation	
0.98	0.01	Diversity	ADL	ADL
0.01	0.24	Diversity	IADL	
0.37	0.09	Diversity	Social participation	
0.94	0.007	Diversity	ADL	Socialization
0.68	0.042	Diversity	IADL	
0.33	0.09	Diversity	Social participation	

**Table3:** Comparison of participation in occupational areas between normally developing and children with physically disability

Significance level	Mean Difference	Mean (SD)		Dimensions	Occupations
		Children with physical disability	Normally developing children		
0.44	0.13	9.91 (1.58)	10.04 (0.63)	Diversity	ADL
0.000	3.27	53.50 (7.95)	56.77 (4.09)	frequency	
0.000	-6.25	18.63 (3.91)	18.63 (3.91)	With whom	
0.000	-4.43	36.31 (8.94)	31.88 (6.87)	Enjoyment	
0.000	2.47	5.67 (2.13)	8.14 (1.62)	Diversity	IADL
0.000	14.51	26.07 (1089)	40.58 (8.59)	frequency	
0.000	3.36	10.47 (3.96)	13.83 (3.4)	With whom	
0.000	6.45	24.36 (9.86)	30.81 (7.23)	Enjoyment	
0.000	3.91	4.92 (2.36)	8.83 (2.26)	Diversity	Play
0.000	17.65	19.11 (12.62)	36.76 (11.14)	frequency	
0.000	9.62	13.46 (5.73)	23.08 (8.05)	With whom	
0.000	16.58	22.73 (11.01)	39.31 (10.82)	Enjoyment	
0.000	3.48	6.87 (2.6)	10.35 (3.29)	Diversity	Leisure
0.000	13.09	24.48 (11.48)	37.57 (12.98)	frequency	
0.000	6.9	13.79 (5.29)	20.69 (7.27)	With whom	
0.000	13.67	31.03 (12.67)	44.7 (14.17)	Enjoyment	
0.000	1.37	4.64 (2.33)	6.01 (2.86)	Diversity	Social participation
0.000	6.64	10.23 (6.61)	16.87 (9.66)	frequency	
0.000	4.49	10.16 (4.98)	14.65 (7.62)	With whom	
0.000	5.2	20.76 (10.9)	20.76 (10.9)	Enjoyment	
0.191	-0.2	1.34 (1.05)	1.14 (1.1)	Diversity	Education
0.787	-0.17	4.84 (3.9)	4.67 (4.9)	frequency	
0.008	-1.33	4.15 (3.81)	2.82 (3.22)	With whom	
0.286	-0.73	5.79 (4.72)	5.06 (4.91)	Enjoyment	
0.000	-0.36	1.36 (0.55)	1 (0.14)	Diversity	Work
0.002	-0.79	6.58 (2.4)	5.79 (0.83)	frequency	
0.000	-1.05	2.64 (1.35)	1.59 (0.62)	With whom	
0.000	-1.85	5.69 (2.98)	3.84 (1.16)	Enjoyment	
0.000	0.52	2.44 (0.55)	2.96 (0.19)	Diversity	Sleep/rest
0.000	3.3	13.99 (3.13)	17.29 (1.75)	frequency	
0.136	0.27	3.85 (1.25)	4.12 (1.28)	With whom	
0.47	0.28	10.57 (2.8)	10.85 (2.67)	Enjoyment	

## Discussion

The purpose of this study was to assess the construct validity and reliability of the CPAS-C scale in 6-12-year-old children with physical disabilities. For assessing the internal consistency, the Cronbach’s alpha coefficient (α) was calculated. The results of this study were consistent with the results of Lim et al. (2018), who reported that the internal consistency of this instrument had a diversion score ranging from 0.59 to 0.92 for the participation scale and a score of 0.73 to 0.79 for the environmental scale (25). In our study, the Cronbach’s alpha scores of the ADL, IADL, play, leisure, and social participation domains were poor to acceptable. The results of this study were consistent with the findings of King et al. (2006) and Rosenberg et al. (2010). Previous studies have shown that participation in life areas depends on various factors, such as the level of disability, culture, lifestyle, living environment, and personal characteristics (28, 29). Therefore, considering that our participants were from different parts of Tehran with different cultures, as well as the fact that they had different diagnoses and levels of disabilities, their variable participation in different activities was not unexpected. 

The reliability of the CPAS-C scale was assessed in two phases with a 2-week interval by holding in-person interviews with 40 children. The results of this study indicated that CPAS-C had acceptable reliability (ICC: 0.6-0.99). The test-retest reliability of the scale was roughly in line with that of the study developing CPAS-C.

In this study, convergent (VABS) and divergent (comparison of two groups) validities were also measured. The results of convergent validity showed no significant correlations between the domains of CPAS-C (i.e., ADL, IADL, and social participation) with their respective VABS domains, which was in line with the results of Amini et al. (2016) on the correlations between CPAS-C and VABS, as well as between CPQ and VABS (12, 15). Therefore, it can be concluded that VABS is not a good tool for assessing the convergent validity of CPAS-C, and more appropriate tools should be used in future studies. To calculate divergent validity, the participation rate was compared between typically developing children and those with PDs, indicating higher participation rates in all occupations in normally developing children than children with PD (P<0.001). Overall, our results showed that CPAS-C had an acceptable divergent validity, which was consistent with the findings of Yeger et al. (2009), Imms et al. (2008), and Majnemer et al. (2008) (5, 26, 27). This could be due to the tiredness of physically-disabled children, the lack of paying attention by the family and community to these children’s activities, the lack of motivation in these children, and the lack of proper feedback for their participation.

## In Conclusion

In this study, the Children's Participation Assessment Scale -Child version that evaluates all eight occupation areas (i.e., ADL, IADL, play, leisure, social participation, education, work, and sleep/rest) delivered acceptable psychometric properties (i.e., construct validity and convergent, divergent, and internal consistencies). Therefore, it can be used as a valid and reliable scale for assessing the participation of 6-12-year-old children with PDs. It can also be utilized as a guide for interventional experiments and a comprehensive framework for clinical evaluations.

## Authors contribution

 Malek Amini, Afsoon Hassani Mehraban: Project Design and supervise. Omid Rostamzadeh: Data gathering, wrote the article. Omid Rostamzadeh, Malek Amini & Afsoon Hassani Mehraban: interpreting and discussing results. All authors agreed to be accountable for all aspects of the work in ensuring that questions related to the accuracy or integrity of any part of the work are appropriately investigated and resolved
